# Molecularly Imprinted Nanoparticles towards MMP9 for Controlling Cardiac ECM after Myocardial Infarction: A Predictive Experimental-Computational Chemistry Investigation

**DOI:** 10.3390/biomedicines10092070

**Published:** 2022-08-24

**Authors:** Anthea Villano, Giovanni Barcaro, Susanna Monti, Niccoletta Barbani, Antonio Rizzo, Daniela Rossin, Raffaella Rastaldo, Claudia Giachino, Caterina Cristallini

**Affiliations:** 1 CNR-IPCF, National Research Council—Institute for Chemical and Physical Processes, Area della Ricerca, Via Moruzzi 1, I-56124 Pisa, Italy; 2 CNR-ICCOM, National Research Council—Institute of Chemistry of Organometallic Compounds, Area della Ricerca, Via Moruzzi 1, I-56124 Pisa, Italy; 3 Department of Civil and Industrial Engineering, University of Pisa, I-56122 Pisa, Italy; 4 Department of Clinical and Biological Sciences, University of Turin, I-10043 Orbassano, Italy

**Keywords:** molecularly imprinted nanoparticles, nanomedicine, therapeutic target, metalloproteinase control, left ventricular remodeling, molecular dynamics, FT-IR Chemical Imaging

## Abstract

The recent advances in nanotechnology are revolutionizing preventive and therapeutic approaches to treating cardiovascular diseases. Controlling the extracellular matrix metalloproteinase (MMP) activation and expression in the failing human left ventricular myocardium represents a significant therapeutic target for heart disease. In this study, we used molecularly imprinting polymers (MIPs) to restore the correct balance between MMPs and their tissue inhibitors (TIMPs), and explored the potential of this technique exhaustively through chemical synthesis, physicochemical and biological characterizations, and computational chemistry methods. By molecular dynamics simulations based on classical force fields, we simulated the early stages of the imprinting process in solution disclosing the pivotal interaction established between the monomers and the MMP9 protein template. The average interaction energies of methacrylic acid (MAA) and poly (ethylene glycol) ethyl ether methacrylate (PEG) units were in the ranges 17–22 and 30–37 kcal/mol, respectively. At low coverage, the PEG monomers seemed firmly anchored to the protein surface and were not displaced by water, while only about 20% of MAA was replaced by water. The synthesis of MIPs was successfully with a monomer conversion higher than 99% and the production of spherical particles with average diameter of 344 ± 33 nm. HPLC analysis showed a specific recognition factor of MMP9 on MIPs of about 1.3. FT-IR Chemical Imaging confirmed the mechanisms necessary to generate a “selective memory” of the MIPs towards the enzyme. HPLC results indicated that the rebound amount of both TIMP1 and MMP2 to MIPs is lower than that of the template, showing a selectivity factor of 2.1 and 2.3, respectively. Preliminary tests on the effect of MIPs on H9C2 cells revealed that this treatment has no cytotoxic effects.

## 1. Introduction

Myocardial infarction (MI) is considered one of the leading causes of death worldwide, being responsible for over 15% of mortality each year [1]. One of the most common complications of MI is left ventricular (LV) remodeling, which is a geometric distortion of the left ventricle derived from changes in the extracellular matrix (ECM). This is caused by the necessity to restore the pump function and hypertrophy of the undamaged myocytes. The left ventricular derangement may induce most ischemic complications, such as late post-ischemic heart failure. All cell types in the myocardium, either in a basal condition (myocytes, fibroblasts, and endothelial cells) or in response to inflammation (macrophages and neutrophils), can typically express one or more types of matrix metalloproteinases (MMPs), proteolytic enzymes that likely contribute to ECM changes and myocardial remodeling. Their essential role in the progression of left ventricular dimension, remodeling, and mortality following MI has been demonstrated in the past decades in several primary and clinical studies [2,3]. The expression of MMPs varies among different physiological and pathological states. For this reason, restoring the correct balance between MMPs and their inhibitors (TIMPs) is essential.

The treatment of MI represents a priority for regenerative medicine to improve the health and the quality of life of many patients. Despite the current pharmacological approaches and therapies used to replace the damaged cardiac tissue, the morbidity and mortality of patients with problems after MI remain very high. Several strategies have been explored to overcome the limits of these treatments. These include genetic modifications and the introduction of bioactive factors and strategies of tissue engineering based on 3D constructs able to provide mechanical support to the damaged myocardial tissue [4,5,6]. The main objective remains the instruction of stem cells, both seeded and in situ recruited, towards a correct morphogenesis of cardiac tissue [7,8]. Various studies demonstrated that scaffolds might regulate stem cell behavior by stimulating their differentiation towards a cardiac lineage [9,10]. However, specific control of the remodeling process at the ECM level is also essential. Pre-clinical and clinical studies demonstrated that MMP9 has a critical pathophysiological significance in cardiovascular disease. Some authors measured the plasma MMP/TIMP levels and LV geometry in patients up to 6 months after MI, showing an increase by >150% of MMP9 from control (13 ± 3 ng/mL) at on day 1 after MI compared to control (47 ± 6 vs. 13 ± 3 ng/mL) and a further n increase by 35% in MMP9 levels from day 1 to day 5 after MI [11]. A similar trend was observed in patients with acute MI [12]. These data are in line with pre-operative value evaluated in patients with myocardial infarction at day 1 [13]. Higher MMP9 correlated with larger LV volumes and greater adverse LV remodeling [11,12]. No significant relationships were observed between early changes in MMP2 and TIMP1 and the degree of LV dilation [11].

Most currently used drugs for heart failure can reduce MMP9 levels. However, only a specific inhibitor strategy targeting MMP9 can be effective [14,15]. In a recent work, pharmacological MMP9 inhibitors, salvianolic acid B and MMP9 inhibitor-I, and clustered regularly interspaced short palindromic repeats (CRISPR)/CRISPR-associated protein 9 (Cas9)-mediated MMP9 genetic ablation were evaluated [15]. Therefore, reduction of MMP9 activity is shown to be an interesting current target strategy in order to prevent the exacerbation of heart failure. The molecularly imprinting nanotechnology has the potential of selectively removing the overexpressed MMP9 [16]. The molecularly imprinting technology (MIT) is a synthesis process that produces tailor-made polymeric materials with memory for a selected template molecule. This technique relies on three crucial steps: the creation of the monomer–template complex, the use of a crosslinker (needed to preserve the interactions), and the removal of the template. The last step uncovers the specialized cavities capable of rebinding the template.

One of the most important applications of MIT is the production of biomimetic sensors using small or large molecules [17,18]. Recently, this technology was applied for the detection of protein markers of cardiovascular disease [19]. In our study, the molecularly imprinted nanoparticles (MIPs) towards the enzyme MMP9 were not proposed as diagnostic tool but for a therapeutic application; however, also in this case, the issues regarding the use of a macromolecule as template need to be considered.

In this context, the formation of MIPs at a low monomer concentration during the pre-polymerization stage is a desirable scenario. This is because the polymerization process is exothermic and can reach non-physiological high temperatures, which could alter the protein secondary structure with the substantial alteration of the MIP mapping and loss of template recognition during rebinding [20]. Furthermore, a relatively low monomer concentration during polymerization can preserve the native protein structure. At high polymer coverage, the entrapment of the protein within the MIP environment can occur with an inherent difficulty in template removal after imprinting. Mass transport in large proteins can hinder the template from reaching far and deep cavities of the MIP, limiting the template rebinding to the surface cavities only. This induced scientists worldwide to develop various surface imprinting methods [20]. For an effective recognition and efficient template binding, the surface cavities should possess highly selective regions that easily accommodate the protein. At low monomer concentration, the template should show preferential areas of adsorption (affinity for the chosen monomers) to avoid a random displacement of the monomers and thus a reduced specificity of the MIP.

Computational molecular modeling is often combined with experiments to describe and predict possible MIP structures assembled for the recognition of small molecules [21,22,23,24,25,26,27]. Indeed, it was capable of providing a wealth of valuable data during the optimization of the recognition process, suggesting preferential routes to the synthesis process. Instead, in the case of protein targets, it was more rarely employed [28,29,30]. The earliest investigations used simple kinetic models to rationalize the fundamental principles of interaction between the imprinted hydrogel and the globular proteins, then the details of the molecular structures were included through granular descriptions (coarse-grained models with the Martini Force Field [31]). These were applied to simulate hydrogels interacting with proteins characterized by different imprinting properties. More recently, atomistic approaches have been used to rationalize the choice of the monomers for maximizing/tuning the interaction with a given protein template [32,33], or with the surrounding species, as in the case of a prostate-specific antigen (PSA) in a proto-polypyrrole environment [34].

In this work, we combined molecular modeling at the atomic level, chemical synthesis, physicochemical and biological characterizations to design, and MIPs production for the efficient recognition and removal of the human MMP9 enzyme. The final application of these systems will be their loading on a medical device to be implanted on the heart, our aim in this work was provide a comprehensive in vitro characterization of these intelligent nanoparticles needed before the in vivo biological evaluation. We defined a pre-polymerization computational protocol based on classical atomistic simulations for identifying the specific regions of accumulation of the selected monomers, which are good candidates to specialize the recognition ability of the formed MIP. In fact, it was shown that the recognition property could be expanded if the template shows preferential regions of monomers’ adsorption. On the contrary, a random organization of the monomers around the template might indicate that the selected species are not the best choice for the chosen target. Here, we demonstrate that this is the case for the investigated gelatinase and the selected monomers. We want to point out that the focus of this investigation is not on the examination of the structural changes of MMP9, its binding sites, the various amino acids involved in binding processes, etc., which were extensively studied in the past [35], but on the protein as a whole, its surface coating, cavities, and the envelopment of the molecule in a dense network of self-interacting monomers, which are precursors of the copolymer. Two water-soluble monomers, namely methacrylic acid (MAA) and poly (ethylene glycol) ethyl ether methacrylate (PEG), were used to build the MIP copolymer in a molar proportion of 75% to 25%, respectively. PEG, although interacting less strongly with the protein, can enhance MIP biocompatibility and prevent inflammatory reactions [36]. A preliminary study showed that the chosen molar proportion of these monomers was suitable for obtaining nanoparticles selective towards MMP9 [16] and the most efficient copolymers. To evaluate the dimensions of MIPs, before and after template removal, morphological analysis (SEM) and dynamic light scattering (DLS) were carried out. A detailed investigation of the complex MMP9/MIPs, before and after template extraction procedure, was carried out using Fourier-transform electron infrared (FTIR) Chemical Imaging, allowing us to evaluate the distribution of MIP components. High-Performance Liquid Chromatography (HPLC) was used in order to assess both the rebinding capacity of MIPs towards MMP9 and the capability of MIPs to discriminate between MMP9 and two different molecules, TIMP1 or MMP2. MMP2 was chosen not only for its similarity to MMP9 in terms of size and functionality, but also because MMP2 and MMP9 are closely related collagenases and involved in the turnover of the ECM after myocardial infarct. Finally, in vitro cell viability using H9c2 cells, at different MIPs doses, was assessed.

## 2. Materials and Methods

### 2.1. Materials

All the materials and reagents were obtained from Sigma-Aldrich (Milan, Italy), unless otherwise specified. Methacrylic acid (MAA) and poly(ethylene glycol) ethyl ether methacrylate ((PEG)EEMA, average molecular weight 246 Da) as monomers, and thrimethylolpropane thrimethacrylate (TRIM) as a crosslinker, were used. Sodium metabisulfite (Na_2_S_2_O_5_) and ammonium persulfate ((NH_4_)_2_S_2_O_8_) were dried and used as radical initiator of the reaction. Matrix Metalloproteinase-9 (MMP9) human recombinant as template, TIMP metallopeptidase inhibitor 1 (TIMP1) human recombinant and Matrix Metalloproteinase-2 (MMP2) human recombinant as analog enzymes, were used. Phosphate-buffered saline (PBS) solution with pH 7.4 was used as the reaction and rebinding medium. Methanol (MeOH), ethanol (EtOH), acetic acid (MeCOOH), and bidistilled water were used for template extraction. Acetonitrile (ACN) purchased from Carlo Erba Reagenti, Cornaredo (MI), Italy, with an HPLC purity degree and bidistilled water were used as HPLC mobile phase.

### 2.2. Computational Methods

Molecular Dynamics (MD) simulations were carried out through the AMBER16 software [37] employing the General Amber Force Field (GAFF) [38,39] for MAA and PEG, and the ff14SB Force Field for the human MMP9 protein [40]. At pH = 7, the protein is negatively charged, and it was neutralized by adding 9 Na^+^ counterions. MAA (pKa ≈ 4.8) should be negatively charged too, but we had to opt for its neutral (COOH) form to avoid the addition of many counterions that could have biased the simulation results. It is worth mentioning that their interactions with the surrounding amino acid residues could be underestimated as hydrogen bonds instead of salt bridges.

MAA and PEG isolated molecules were firstly optimized at the DFT level with the use of Gaussian09 [41] by employing the B3LYP hybrid functional [42] and the 6-31G(d) basis set. Atomic charges were determined with the Restrained electrostatic potential atomic charges (RESP) procedure by fitting them to the B3LYP/6-31G(d) electrostatic potential in the space surrounding the molecules with hyperbolic restraints on the non-hydrogen atoms.

At each monomer’s content, the system was first equilibrated at 300 K, first in the NVT and then in the NPT ensemble, and then sampled through MD production runs of variable length, according to the absence or presence of solvent molecules (around 1 ns in the first case and 12–14 ns in the second case). In all the simulations, the Berendsen thermostat and barostat were used [43] to maintain temperature and pressure. The analyses were carried out by using the AmberTools of the AMBER16 package and the Chimera software [44].

### 2.3. Synthesis of Imprinted Nanoparticles

MMP9/MIPs were synthesized using the precipitation polymerization method in diluted monomer concentration. The synthesis was performed by radical polymerization of MAA and (PEG)EEMA, with monomer composition 75/25 mol/mol, in the presence of TRIM (20% respect to monomer). Human MMP9, also known as gelatinase B, was used as a template and added in the PBS reaction feed at 50 ng/mL concentration. After equilibrating the reaction feed at 37 °C, and degassing with nitrogen, the redox couple initiator was finally added under mild agitation. After 120 min from the reaction initiation, the product was recovered and washed to remove the unreacted monomers or crosslinker and free enzyme. The extraction procedure of the reaction product was performed using different sequential solutions under constant stirring: MeCOOH solution (0.1% *v*/*v* in water) and MeOH blend (30/70) solution, EtOH/bidistilled water (70/30) blend, and pure bidistilled water. Non-imprinted nanoparticles (CPs) were prepared as a control in the absence of MMP9 and treated in the same way.

### 2.4. Characterization of Imprinted Nanoparticles

Morphological and dimensional analysis was performed using scanning electron microscope (SEM, FEI QuantaTM 450 FEG Instrument, Hillsboro, OR, USA). The MIPs and CPs were sputtered with gold to improve the quality of the analyses. SEM images were analyzed with the open-source ImageJ software. Results were expressed in terms of Z-average values and standard deviation, considering at least 100 single particles per sample. Zetasizer Nano ZS90 Dynamic Light Scattering (DLS, Malvern Instruments, Malvern, UK) was used to evaluate the Z-average diameter of NPs after their dispersion in EtOH/bidistilled water to improve the disaggregation of NPs. For each sample, a vial containing a NP dispersion was placed into a water bath sonicator for 20 min at high frequency. High-Performance Liquid Chromatography (HPLC Perkin Elmer Series 200) was used to evaluate the amount of MMP9 in the reaction feed in the extraction and rebinding solutions. Rebinding tests were performed by placing MIPs and CPs in the presence of rebinding solution (10 ng of MMP9 in 1 mL of PBS) under dynamic conditions. At prefixed times, the whole volume of rebinding solution is withdrawn and ultra-centrifuged. To evaluate MMP9/MIP selectivity, rebinding solutions containing analog enzymes, TIMP1 and MMP2, were put in contact with MIPs. The supernatant is analyzed by HPLC to evaluate the residual amount of MMP9, MMP2, and TIMP1. A C18-Synergy Hydro-RP (Phenomenex, S.r.l., Castel Maggiore, Bologna, Italy) column was used with ACN/water (80/20 *v*/*v*) as mobile phase (flow rate 0.8 mL/min). UV detector was set at 280 nm, and injections were performed by auto-sampling 100 μL of different solutions. Chemical analysis on pure MMP9, on particle product (MIPs before and after MMP9 extraction and CPs) was carried out by FT-IR Chemical Imaging (Perkin Elmer Spotlight 300, Shelton, CT, USA) in order to obtain chemical and correlation maps allowing us to evaluate the MMP9/MIPs interactions. Spectral images were acquired in attenuated total reflectance (μATR) mode (spectral resolution of 4 cm^−1^). The data analysis was performed by Principal Component Analysis (PCA).

Cytocompatibility analyses were performed on MIPs over a wide range of doses using the Propidium Iodide Flow Cytometry assay and H9C2 cardiomyoblasts. H9C2 cells were seeded in a 24-well plate. In total, 5 × 10^4^, 4 × 10^4^ and 2.5 × 10^4^ cells per well were seeded for the 24, 48, and 72 h exposure periods, respectively. Cells seeded alone were used as a negative control. Then 24 h after seeding, the culture medium was removed from each well and replaced with 800 μL of fresh culture medium and 200 μL of sterile-filtered MIP extract to reach the final concentrations of 0.64, 6.4, 12.8, and 64 mg/mL. After 24, 48, and 72 h of culture, cells were detached with Trypsin-0.2% EDTA, resuspended in 500 μL of cold 1X PBS (Sigma-Aldrich), and transferred into falcon tubes for flow cytometry analysis. Then 10 μL of Propidium Iodide (1:20 in bidistilled water, Sigma-Aldrich) was added, and after 5 min, cell viability was analyzed through flow cytometry. The experiments were carried out twice, in triplicates for each condition.

## 3. Results and Discussion

**Figure 1:**
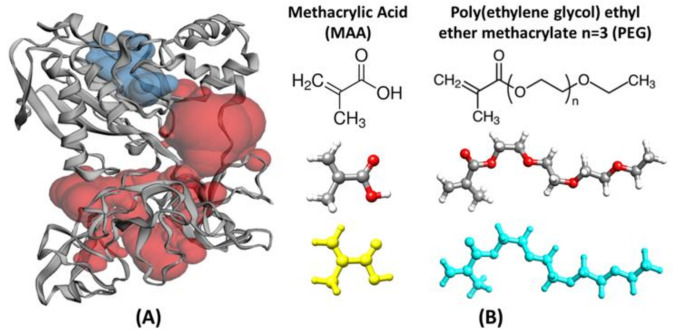
(**A**) Structure of the MMP9 protein extracted from PDB; the ribbon representation of the protein secondary structure is combined with a visualization of the two most significant pockets identified by the CASTp web program [45]. These are the catalytic domain (blue spheres) and the central region of the protein between the catalytic + propeptide domain and the FnII domains; (**B**) structures of the MAA and PEG monomers.

To give an idea of the leading forces driving the monomers towards the protein template, we used preliminary molecular dynamics simulations focusing first on the effects of the monomer concentration. Then, we extended the simulations to reproduce a possible polymerization process in solution. The purpose was to evaluate the dynamic competition between water and the monomers for the active adsorption sites on the protein surface and identify the regions of preferential adsorption (accumulation) of the monomers during a possible pre-polymerization phase. The MMP9 protein structure chosen for these calculations (structure 1l6j [35] downloaded from http://www.rcsb.org in 1 September 2020) (Figure 1) is a proform of the human matrix metalloproteinase MMP9 solved at 2.5 Å resolution. It contains 405 amino acid residues, 2 Zn^2+^, and 3 Ca^2+^ ions and includes the prodomain, the catalytic, and three FnII (fibronectin type II) domains. All these domains, pockets, and clefts have specific binding roles, variable size, definite secondary structure, hydrophobic (FnII domains), and hydrophilic character, and can host water molecules as well. Thus, it is challenging to identify the most appropriate combination of monomers to design a finely tuned MIP.

**Figure 2:**
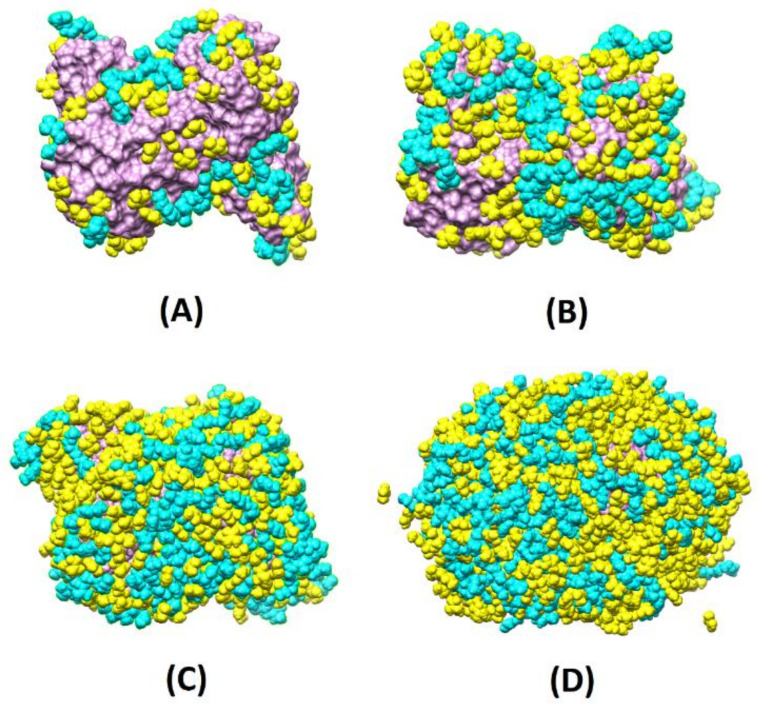
Representative absorption configurations extracted from equilibrated MD simulations of the four scenarios without water described in the main text. (**A**) X concentration, 92 MAA and 26 PEG; (**B**) 2X concentration, 184 MAA and 54 PEG; (**C**) 4X concentration, 368 MAA and 115 PEG; (**D**) 8X concentration, 742 MAA and 236 PEG. MAA and PEG are yellow and cyan, respectively; MMP9 protein is purple.

To evaluate the monomer–water competition, we created four scenarios with different monomer concentrations around the protein (labeled 8X, 4X, 2X, and X, where X is a sub-monolayer regime, consisting of 92 MAA and 26 PEG) with different simulation boxes (8X: 98 × 127 × 105 Å^3^; 4X: 84 × 108 × 85 Å^3^; 2X: 89 × 82 × 76 Å^3^; X: 81 × 100 × 63 Å^3^) and number of waters (8X: around 26,000; 4X: around 15,000; 2X: around 11,000; X: around 10,000) and simulated the migration of the various species towards the surface both without water (1 ns MD runs) and with water (12 ns MD runs). At each concentration, the ratios between MAA and PEG were in the range 3.1–3.5 (more specifically, 8X: 3.1; 4X: 3.2; 2X: 3.4 and X: 3.5), satisfactorily in line with the experimental molar proportion of 75% to 25%. These concentrations corresponded to partial and total coverage of the surface template (reached at 4X, 8X). Inspection of Figure 2, where the final configurations without water are depicted, reveals that already at 4X, the protein surface is almost entirely covered with the monomers, and at 8X, part of the monomers is found in the second layer far from the template interface. Instead, at 2X, the MMP9 surface is partially covered.

**Figure 3:**
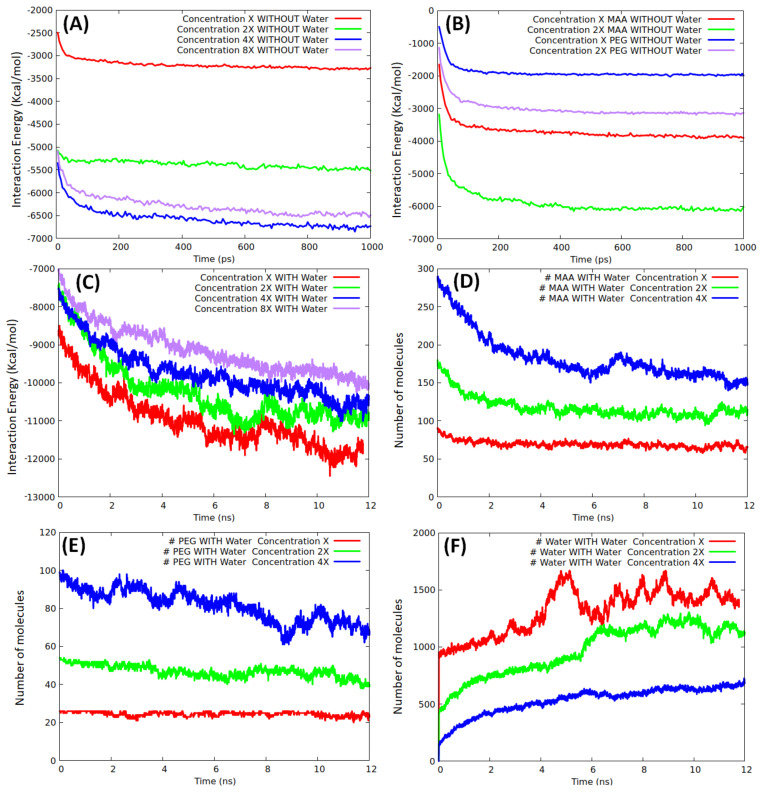
Interaction energy between the protein and the monomers (kcal/mol) as a function of simulation time: (**A**) total, without water; (**B**) MAA and PEG, without water; (**C**) total with water. Evolution of the number of the adsorbed molecules in water within 3.5 Å from the protein surface: (**D**) MAA, (**E**) PEG, and (**F**) water.

Molecular dynamics simulations at ambient temperature were used to provide not only a qualitative picture of the systems, but also a quantitative estimation of the interaction energy of the protein surface with its environment:

E_int_ = E_total_ − E_MMP9_ − E_environment_ (1)

where E_total_, E_MMP9_, and E_environment_ correspond to the energies of the whole system, isolated protein, and environment (made of only monomers or of both monomers and water), respectively. For each monomer concentration, three independent simulations were carried out to sample different interaction scenarios, and the plots shown in Figure 3 are the average over the runs.

A total production run (1 ns) without water was sufficient to reach a stationary state, as confirmed by the fast-converged values of the interaction curves displayed in Figure 3A. At both low and high coverage, we did not observe any appreciable modifications of the protein structure (the root mean square deviation of the backbone atoms of the final geometries with respect to the initial geometry was <1.5 Å), in agreement with the results obtained in similar conditions by Mazouz et al. [34] when simulating a PSA interacting with a proto-polypyrrole environment. As expected, the interaction energy increased when passing from X to 2X, whereas an almost equal (saturation) value was reached at both 4X and 8X, confirming that full coverage was already achieved at 4X. By taking the final interaction energy at 4X/8X as a reference, we tried to give an estimate of the coverage at the other densities finding that 2X roughly corresponded to 80%, whereas X to 40% only. Non-linear effects in the interaction energy trends were due to the competition between inter-monomer and monomer/template components. To evaluate the average interaction energy of the two types of monomers, we investigated them separately, at concentrations of X and 2X without water. The production analysis over an analogous time window (1 ns), shown in Figure 3B, indicates that only at X concentration all the monomers were in contact with the template. The average interaction energies of MAA and PEG units were in the ranges 17–22 and 30–37 kcal/mol, respectively. The interaction energy of the pure monomers reasonably reproduced (underestimate ≈ 10%) the interaction energies corresponding to the mixed systems at both X and 2X concentrations.

**Figure 4:**
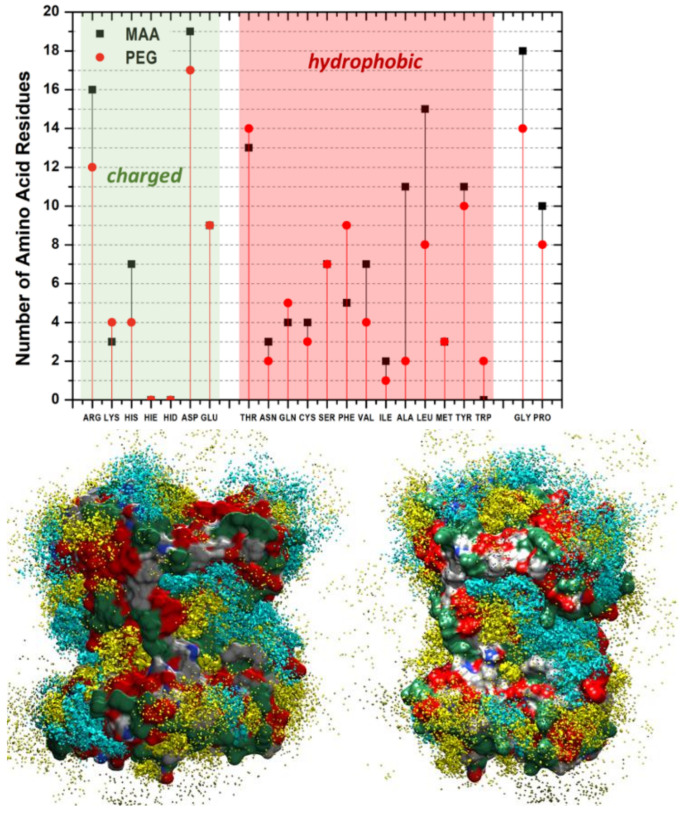
(Upper panel) number of amino acids within 3.5 Å of the adsorbed monomers; (lower panel) SDF (spatial distribution functions) of the MAA and PEG monomers around the MMP9 protein at X concentration with water molecules (undisplayed). MAA yellow and PEG cyan. Contour plots at 0.5 a.u. Red and green regions on the MMP9 surface indicate hydrophobic and charged zones, respectively.

Moving to the mixed model’s simulations in solution, the evolution of the template/environment interaction energies is displayed in Figure 3C. Due to the slow dynamics of water penetration inside the MAA/PEG shell covering the protein surface (thick in the case of the higher concentrations), we had to extend the production run to 12 ns to reach convergence. It is apparent (Figure 3C) that the presence of water molecules is detrimental for the monomers–template interaction. In fact, the interaction energy of the protein with the surrounding environment is dominated by solvent adsorption. This is confirmed by the evolution of the number of MAA (Figure 3D), PEG (Figure 3E), and water molecules (Figure 3F) at X, 2X, and 4X concentrations. At each concentration, water shows the tendency to penetrate within the monomer shell and reach the protein surface by displacing and replacing both MAA and PEG molecules. The effect is weaker at X concentration, where only about 20% of MAA, and almost no PEG molecules are replaced by water. This is because, in this case, a significant portion of protein surface is already available for the adsorption of water molecules (Figure 2A). Instead, at higher concentrations as 4X, the effect becomes more marked, and almost 50% of MAA and 30% PEG units are desorbed. However, in all cases, MAAs are more easily replaced by water than PEGs, probably because of their smaller dimensions. These findings suggest that the high coverage scenario in aqueous media is quite improbable. The examination of the distribution of the monomers around the protein and their most probable locations through spatial distribution functions (SDF) at low coverage (Figure 4) revealed a sort of accumulation in the central region of the enzyme identified by the CASTp program [45] (Figure 1—red spheres). The PEG monomers seemed firmly anchored to the protein surface and were not displaced by water. We could speculate that that portion of the protein is an initial anchoring site and, probably, the starting location of the polymerization process. A mapping of the MMP9 residues located in this region revealed that they are tendentially hydrophobic (Figure 4), and the interactions with PEGs are dominant.

**Figure 5:**
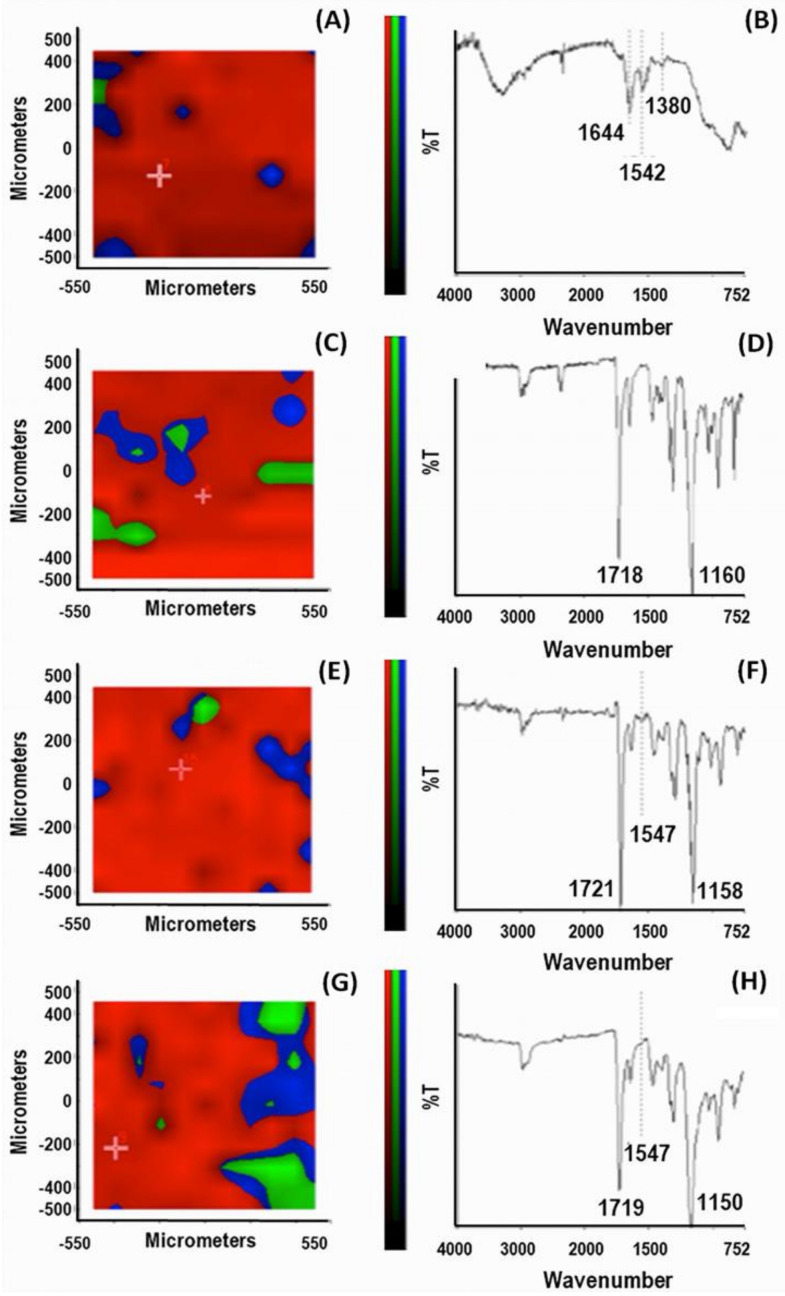
FT-IR Chemical Imaging analysis. PCA map and corresponding spectrum: (**A**,**B**) pure MMP9 after casting the enzyme aqueous solution; (**C**,**D**) CPs; (**E**,**F**) MIPs before MMP9 extraction; (**G**,**H**) MIPs after MMP9 extraction.

On the experimental side, the synthesis was carried out in an aqueous solution at low monomer concentrations (with a molar proportion MAA/PEG of 75%/25%). A crosslinker agent and a couple redox, as an initiator, were added to the polymerization batch to produce NPs in the presence of MMP9. Similarly, control NPs (CPs) were produced and treated with no enzyme addition in the reaction feed. The HPLC analysis established the complete conversion of the monomers (higher 99%) for both MIPs and CPs. The percentage of the enzyme entrapped in the MIPs was about 91.2%, and the amount of enzyme removed after the extraction process was approximately 58.3%. The FT-IR Chemical Imaging was performed to evaluate the chemical map of the NPs aggregates (Figure 5).

First, a chemical map in μATR mode was acquired on the pure MMP9 after casting the MMP9 aqueous solution. Figure 5A shows the PCA analysis. The spectra were acquired in the region with the highest score percentage (red zone) of the total spectral variance; the predominance of the red region in all the examined samples (panels C, E, G) indicates a good chemical homogeneity of the particle aggregates. The spectrum from the PCA map reported in Figure 5B shows the bands corresponding to the protein. Indeed, the absorption of the protein backbone is apparent from the amide I vibration, absorbing at 1644 cm^−1^, the amide II mode at 1542 cm^−1^, and the amide III mode at about 1380 cm^−1^ [46].

**Figure 6:**
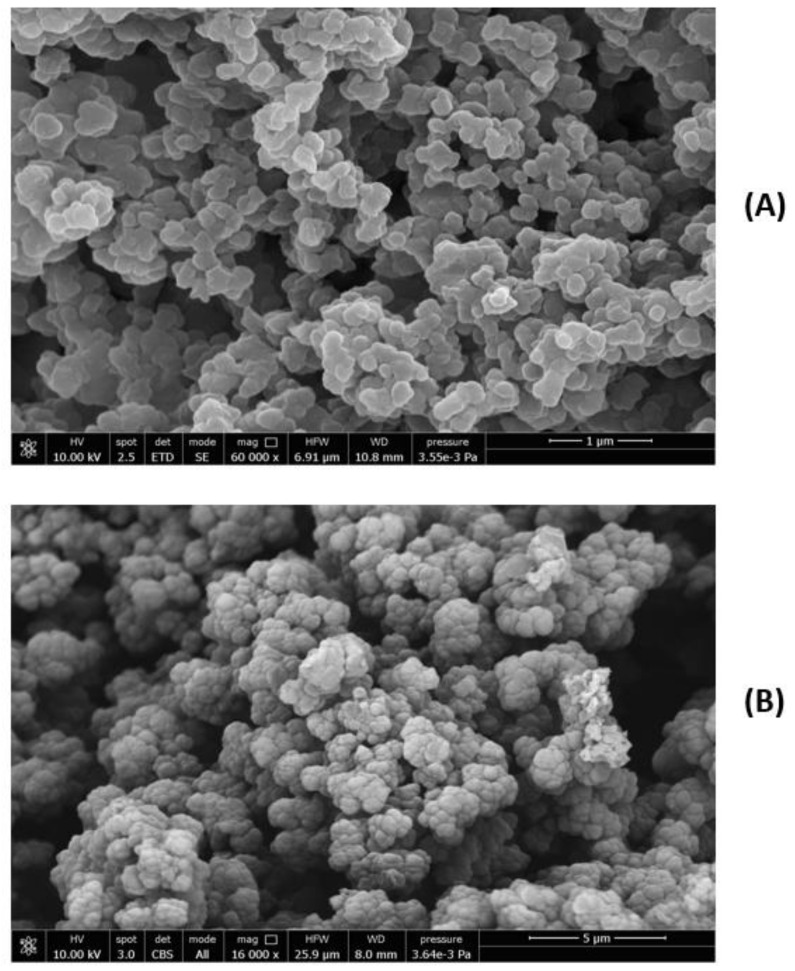
SEM images of (**A**) CPs and (**B**) MIPs.

The identification of the diagnostic band of the enzyme that confirmed its presence in the MIPs was instead challenging due to the overlapping signals of the various species. However, we could recognize the band corresponding to amide II mode at 1547 cm^−1^, as a possible diagnostic reference. Considering the X-ray structure of MMP9 [35], where the active site zinc of the catalytic domain is coordinated with three histidines (His) and the amino acids Asp185 and Glu208 in the calcium-binding sites are highly conserved, we can speculate that a significant contribution to this band can be ascribed to ν(C=C) and ν_as_(COO^−^), of His and Asp185 or Glu208, respectively [46]. The spectrum of CPs shows an intense band with a maximum at 1718 cm^−1^ and a strong band at around 1160 cm^−1^ generated by the stretching of the -C=O and -C-O groups of the PMAA, PEG, and TRIM components. In contrast, no signal in the range of the amide II mode is visible in the spectrum of Figure 5D. In the MIPs spectra, before (panel F) and after the template extraction (panel H), the signal at 1547 cm^−1^ is detectable. A detailed investigation of the complex MMP9/MIPs before the template extraction was carried out. The chemical map detects the presence of the enzyme at the level of the MIP surface. Indeed, the corresponding PCA (panel E) indicates a relatively homogenous distribution of component spectra, including MMP9, over the whole sample. After the template extraction, the band at 1547 cm^−1^ is reduced but still visible, indicating a residual enzyme content. As far as the morphological analysis is concerned, the SEM images of MIPs and CPs are shown in Figure 6A,B.

The tendentially spherical NPs appear agglomerated, but it was possible through the ImageJ software to single out the particles, showing sub-micrometric dimensions. The CP average diameter was 291 ± 70 nm, and the MIP average diameter was 344 ± 33 nm. The size of the single particles shows an average value slightly higher for MIPs after extraction relative to CPs, indicating a modest influence of the template on the MIP dimension. MIPs and CPs after dispersion in EtOH and sonication were also analyzed by DLS. In EtOH, the MMP9 molecules tend to aggregate, giving a value of 204 ± 40 nm, and the size of the MIPs before the extraction is about 988 ± 100 nm. After the extraction, the Z-average value of MIPs is around 750 ± 100 nm, and a value of 700 ± 60 was obtained for CPs. These results indicate that the enzyme was efficiently removed from the MIPs, since the value obtained after the template extraction is smaller than that before extraction, confirming the hypothesis that the formed surface cavities by imprinting do not limit the template removal. Furthermore, considering the DLS values obtained for MIPs (after the extraction) and CPs, we can speculate that the residual immobilized enzyme after the MIP removal can be located among the nanoparticles, not completely separated from them, rather than in deeper sites, as would occur in the case of complete coverage.

In this view, the surface cavities are available for template rebinding in specific regions that can accommodate the enzyme as confirmed by the recognition factor evaluated by HPLC (Table 1).

The adsorption dynamics of MMP9 on MIPs and CPs were studied by measuring the values of Q_t_ (μg of species rebound with respect to grams of NPs) vs. time (data not reported). The adsorption rates, controlled by intermolecular forces, such as hydrogen bonds and electrostatic interactions, rapidly reached the equilibrium for all the NPs. The ratio between the equilibrium rebinding capacity Q_e_ (μg/g) of protein on MIPs and that measured on CPs was 1.3. Thus, the maximum value of Q_t_ was obtained in the case of specific and selective interactions between the template and MIPs due to the presence of the recognition sites. The regions of monomer accumulation, which could be seen as polymerization seeds and recognition zones, were also predicted by the simulations, thus confirming the validity of the proposed computational protocol. To evaluate the selectivity of the MIPs imprinted towards MMP9, these NPs were placed close to the TIMP1 inhibitor and a different ECM metalloproteinase (MMP2). HPLC results indicated that the rebound amount (Q_e_) of both TIMP1 and MMP2 to MIPs is lower than that of the template, showing a selectivity factor of 2.1 and 2.3, respectively. The amount of TIMP1 or MMP2 re-bonded to MIPs (15.2 or 14.1%, respectively) is lower than that of the template re-bonded to MIPs (32.2%). These results indicate the capability of the MIPs to specifically discriminate between MMP9 and analog enzymes (Table 1). In particular, the structure of MMP9 is quite similar to the structure of MMP2 except for the location of fibronectin like-domains [35].

This result is significant in view of a possible MIP application at the level of infarcted cardiac ECM where a selective removal of MMP9 is expected, considering the over-expression of this enzyme after MI, differently from the inhibitor (TIMP1) or other MMPs, like MMP2, whose changes in the ECM after MI are observed but in a less relevant amount than for MMP9. For example, the plasma MMP9 levels measured one day after MI were about 200% higher than the control values. On the contrary, the MMP2 levels were lower than the reference control values [11]. The plasma TIMP1 levels were higher than the control levels at the same endpoint, but the ratio MMP9/TIMP1 increased by a factor of two [11].

**Figure 7:**
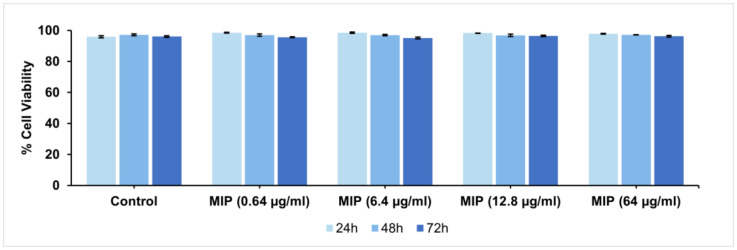
Cytocompatibility analyses of MIPs with H9C2 at 24, 48, and 72 h, evaluated by propidium iodide flow cytometry (mean ± SD; MIPs: n = 6). Negative control: cells seeded without nanoparticles.

Finally, the biocompatibility of the produced MIPs is of fundamental importance for in vivo applications. Cytocompatibility analyses were performed on MIPs over a wide range of doses using the Propidium Iodide Flow Cytometry assay and H9C2 cardiomyoblasts, a valuable in vitro model for cardiac disease studies. Specifically, extracts of MIPs at three different concentrations were added to H9C2 cells, and the viability after 24, 48, and 72 h of exposure was evaluated (Figure 7). The results indicated optimal cell viability at all doses tested and for all three evaluation time-points.

## 4. Conclusions

In this work, molecular imprinting was proposed for the production of smart nanoparticles capable of modulating enzymes of the MMP family for restoring the correct MMPs-TIMPs ratio in the cardiac microenvironment. The molecular dynamics simulations of the monomer’s adsorption and accumulation in specific regions of the MMP9 surface showed that this protein is a suitable template for the MIP formation and that PEG monomers were preferred to MAA in the employed model. This suggests that specific recognition zones can be obtained in the prepared MIPs, as confirmed by a functional analysis towards both the MMP9 inhibitor (TIMP1) and another enzyme similar to MMP9 (MMP2). The characterization of PEG MIPs demonstrated that they have the potential for avoiding left ventricular remodeling after myocardial infarction.

## Figures and Tables

**Table 1:** Recognition and selectivity values of MIPs.

Rebound MMP9 Fraction to MIPs (%)	Rebound MMP9 Fraction to CPs (%)	Rebound TIMP1 Fraction to MIPs (%)	Rebound MMP2 Fraction to MIPs (%)
32.2	24.5	15.2	14.1
	Recognition Factor	Selectivity Factor	Selectivity Factor
	1.3	2.1	2.3

## Data Availability

Not applicable.
